# Validation of a novel iPhone application for evaluating near functional visual acuity

**DOI:** 10.1038/s41598-022-27011-2

**Published:** 2022-12-26

**Authors:** Akiko Hanyuda, Miyuki Kubota, Shunsuke Kubota, Sachiko Masui, Kenya Yuki, Kazuo Tsubota, Kazuno Negishi

**Affiliations:** 1 Department of Ophthalmology, Keio University School of Medicine, 35 Shinanomachi, Shinjuku-Ku, Tokyo, 160-8582 Japan; 2 Epidemiology and Prevention Group, Center for Public Health Sciences, National Cancer Center, Tokyo, Japan; 3 Department of Ophthalmology, Shonan Keiiku Hospital, Kanagawa, Japan; 4 Graduate School of Median and Governance, Keio University, Kanagawa, Japan; 5 Hazawa-Kubota Eye Clinic, Kanagawa, Japan; 6 Tsubota Laboratory, Inc., Tokyo, Japan

**Keywords:** Diagnosis, Health services, Public health, Quality of life, Medical research, Outcomes research

## Abstract

Monitoring dynamic changes in near vision is important for early detection of presbyopia. This study assessed the accuracy and reliability of a new smartphone-based application, the Smart Vision Check (SVC), compared with those of a conventional device (AS-28; Kowa, Aichi, Japan), for measuring near functional visual acuity (NFVA). We enrolled 115 healthy volunteers aged ≥ 20 years with bilateral best-corrected visual acuity of ≥ 20/25. The SVC was designed for use on an Apple iPhone SE2 to measure NFVA by tapping on the orientation icon manually. Conventional FVA was measured using the AS-28 with − 2.50 D added to the best distance correction at baseline. There was no significant difference in NFVA-related measurements between the AS-28 and SVC (P > 0.05). The Spearman correlation coefficients of NFVA measurements between the two devices were over 0.60 (P < 0.001). The Bland–Altman plot indicated minimal bias with limits of agreements of ± 0.34 logMAR for NFVA with habitual correction when comparing the AS-28 and SVC. The intraclass correlation coefficient of the repeated SVC-measured NFVA was 0.915 (95% CI 0.800–0.969). In summary, the SVC has the potential to evaluate NFVA in a relatively easy manner. Applied clinically, the SVC can be useful for presbyopia screening.

## Introduction

Uncorrected presbyopia, an age-related loss of accommodation, is the leading cause of vision impairment globally, with a projected prevalence of 1.8 billion worldwide in 2050^1,2^. Vision impairment from uncorrected presbyopia predominantly affects those in rural areas of low-resource countries, where the residents encounter substantial barriers to healthcare services^2,3^. In fact, the prevalence of presbyopia is highest in the Latin American region, reaching 90% in those aged ≥ 35 years^3^. Furthermore, presbyopia is likely to be undiagnosed globally among younger generations^4^. Even in developed countries with adequate resources and less health inequality, working-age populations are generally too busy to visit ophthalmic clinics for a vision test, or they were unaware of presbyopic symptoms due to the lack of knowledge. In our recent survey, approximately 20% of people aged ≥ 45 years have uncorrected presbyopia, despite having difficulty in near vision tasks^5^. A report from rural China has shown that one third of the participants (28.8%) without near correction did not recognize their presbyopic condition^6^. Considering that wearing glasses can successfully correct presbyopia, thereby retaining the workforce^7^, there are unmet public health and clinical needs for early detection and correction of presbyopia.

Functional visual acuity (FVA) has been increasingly recognised as a good proxy for visual performance in daily life^8^. FVA testing measures the averaged VA during a certain time frame (rather than the VA at a specific point), thereby enhancing the minutest ocular surface abnormality and vision-related quality of life, which are undetectable by conventional VA testing^9^. Indeed, a clinical study revealed that compared to conventional near VA, near FVA (NFVA) had a significantly stronger correlation with decreased accommodation in eyes with early presbyopia^10^. Given that uncorrected presbyopia leads to a significant productivity loss and decreases self-esteem due to eye strain and asthenopia^3^, timely monitoring of NFVA, similar to other physical parameters such as blood pressure or body weight, is inevitable to maintain visual health.

Smartphones and other wireless technologies are increasingly ingrained into clinical practice^11–13^. In 2019, approximately 70% of the global population used smartphones^14^, with 204 billion applications being downloaded worldwide^15^. The coronavirus disease 2019 (COVID-19) pandemic illustrates the substantial role of mobile applications in reducing health disparities and implicit bias as well as gathering massive real-time datasets with relatively low-cost computing resources^16^. Given the increasing need for capturing daily visual function in an affordable manner, we have developed a novel smartphone application, the Smart Vision Check (SVC), under consignment contract with Keio University Global Research Institute/IoT Healthcare Research Consortium. The SVC may particularly help identify working-age populations who have uncorrected presbyopia but remain undiagnosed by conventional VA testing or those with limited access to ophthalmic clinics.

The aim of this study was to evaluate the accuracy and reliability of the SVC for measuring NFVA, compared with those of a conventional device, to provide a basis for further research involving its use in clinical practice and home settings.

## Results

### Baseline characteristics of the study participants

**Figure 1:**
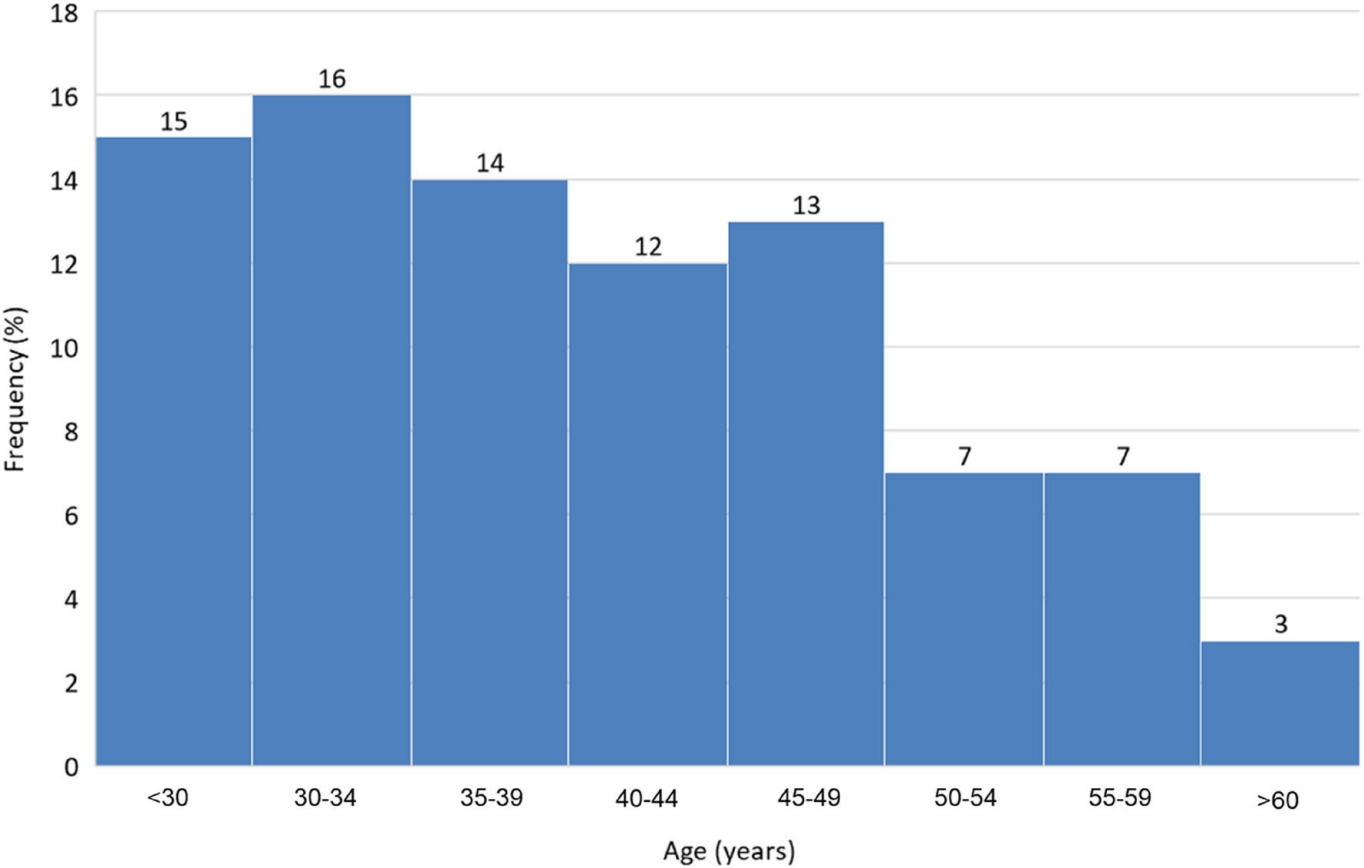
Age distribution of the study participants.

We enrolled 115 participants (73 [63.5%] men and 42 [36.5%] women), with a mean age of 42.5 ± 10.8 years (Fig. 1). Table 1 shows the baseline characteristics of the study participants. The mean monocular subjective refraction (spherical equivalent) and objective refraction of the right eye were − 3.55 ± 3.58 and − 3.81 ± 3.62 D, respectively. The mean monocular corrected distance VA (CDVA) and distance-corrected NVA (DCNVA) at 40 cm were − 0.16 ± 0.05 and 0.04 ± 0.22, respectively. The mean binocular DVA with habitual correction (DVAHC) and NVA with habitual correction (NVAHC) at 40 cm were − 0.12 ± 0.10 and − 0.07 ± 0.12, respectively. The mean tear break-up time (TBUT) was 6.16 ± 2.74 s, and the mean ocular surface staining (OSS) score was 0.11 ± 0.30, with no signs of severe dry eye disease in any of the participants.

**Table 1:** Baseline demographic and clinical features of the study participants.

Baseline characteristics^a^	All (n = 115)
Age, years	42.5 (10.8)
Men, n	73.0 (63.5)
**Monocular examination (right eye)**	
Subjective refraction (SE), D	− 3.55 (3.58)
Objective refraction (SE), D	− 3.81 (3.62)
CDVA, logMAR	− 0.16 (0.05)
DCNVA at 40 cm, logMAR	0.04 (0.22)
TBUT, s	6.16 (2.74)
Ocular surface staining, score	0.11 (0.30)
Amplitude of accommodation	2.32 (2.23)
Distance pupillary diameter, mm	5.34 (0.82)
Near pupillary diameter, mm	4.36 (0.98)
**Binocular examination**	
CDVA, logMAR	− 0.17 (0.03)
DVAHC, logMAR	− 0.12 (0.10)
DCNVA at 40 cm, logMAR	− 0.02 (0.18)
NVAHC at 40 cm, logMAR	− 0.07 (0.12)

^a^Values are presented as means (SD) for continuous variables and percentages for categorical variables.

*CDVA* corrected distance visual acuity, *D* dioptres, *DCNVA* distance-corrected near visual acuity, *DVAHC* distance visual acuity with habitual correction, *logMAR* logarithm of the minimum angle of resolution, *NVAHC* near visual acuity with habitual correction, *SD* standard deviation, *SE* spherical equivalent, *TBUT* tear break-up time.

### Differences in near functional visual acuity measurement between the AS-28 and the Smart Vision Check

We compared the NFVA with habitual correction and distance-corrected NFVA between the conventional AS-28 and the SVC techniques. There was no significant difference between the AS-28 and the SVC in any of the five NFVA-related parameters (Table 2). The average NFVA with habitual correction and distance-corrected NFVA were 0.06 ± 0.22 and 0.12 ± 0.24 for the AS-28, and 0.04 ± 0.13 and 0.10 ± 0.20 for the SVC, respectively, with no significant difference (P > 0.05) (Table 2). In the secondary analyses, we compared NFVA with habitual correction and distance-corrected NFVA as measured using the AS-28 vs. the SVC according to age, sex, TBUT, use of contact lenses, and NVA (Supplementary Table S1). The average VA was significantly greater when measured by the AS-28 than by the SVC among those with an NVA of < − 0.10 logarithm of the minimum angle of resolution (logMAR) compared with those with an NVA of ≥ − 0.10 logMAR. The mean NFVA with habitual correction was − 0.15 ± 0.02 when measured with the AS-28 and − 0.06 ± 0.07 when measured with the SVC (P < 0.001), whereas the distance-corrected NFVA was − 0.15 ± 0.02 when measured with the AS-28 and − 0.06 ± 0.05 when measured with the SVC (P < 0.001) (Supplementary Table S1). However, there was no significant difference in the average VA between the AS-28 and the SVC when stratified by sex, TBUT, or use of contact lenses (Supplementary Table S1).

**Table 2:** Measurement of near functional visual acuity (NFVA) by the AS-28 and the Smart Vision Check (SVC).

Parameters	AS-28	SVC	*P* value^¶^
**NFVA with habitual correction**			
Average VA	0.06 (0.22)	0.04 (0.13)	0.26
Maximal VA	− 0.06 (0.18)	− 0.07 (0.11)	0.49
Minimal VA	0.20 (0.28)	0.18 (0.20)	0.27
Average response time	1.29 (0.14)	1.29 (0.12)	0.88
VMR	0.94 (0.08)	0.93 (0.05)	0.08
**Distance-corrected NFVA**			
Average VA	0.12 (0.24)	0.10 (0.20)	0.15
Maximal VA	0.00 (0.21)	− 0.03 (0.15)	0.07
Minimal VA	0.30 (0.33)	0.26 (0.28)	0.12
Average response time	1.28 (0.16)	1.28 (0.12)	0.93
VMR	0.93 (0.10)	0.92 (0.07)	0.51

The NFVA was evaluated by five parameters, including average, maximal, and minimal VAs, average response time, and VMR. The VMR is the ratio of FVA divided by the value of baseline VA: VMR = (lowest logMAR VA score − logMAR FVA)/(lowest logMAR VA score − baseline logMAR VA). All VA measurements were evaluated in logMAR.

^**¶**^Paired t-test.

*logMAR* logarithm of the minimum angle of resolution, *NFVA* near functional visual acuity, *SVC* Smart Vision Check, *VA* visual acuity, *VMR* visual maintenance ratio.

### Correlations of near functional visual acuity measurement between the AS-28 and the Smart Vision Check

The Spearman correlation coefficients between NFVA as measured using the AS-28 and the SVC are summarised in Table 3. In both NFVA with habitual correction and distance-corrected NFVA, all NFVA-related parameters in the two different techniques were significantly correlated with each other (P < 0.001). Specifically, we observed a high correlation for the average, maximal, and minimal VA. The Spearman correlation coefficients of the average, maximal, and minimal VA were 0.62 (P < 0.001), 0.60 (P < 0.001), and 0.56 (P < 0.001), respectively, for NFVA with habitual correction; and 0.66 (P < 0.001), 0.65 (P < 0.001), and 0.62 (P < 0.001), respectively, for distance-corrected NFVA.

**Table 3:** Correlation of near functional visual acuity (NFVA) between the AS-28 and the Smart Vision Check (SVC).

Parameters	*r*^¶^	*P* value
**NFVA with habitual correction**		
Average VA	0.62	< 0.001
Maximal VA	0.60	< 0.001
Minimal VA	0.56	< 0.001
Average response time	0.34	< 0.001
VMR	0.40	< 0.001
**Distance-corrected NFVA**		
Average VA	0.66	< 0.001
Maximal VA	0.65	< 0.001
Minimal VA	0.62	< 0.001
Average response time	0.31	< 0.001
VMR	0.34	< 0.001

The NFVA was evaluated by five parameters, including average, maximal, and minimal VAs, average response time, and VMR. The VMR is the ratio of FVA divided by the value of baseline VA: VMR = (lowest logMAR VA score − logMAR FVA)/(lowest logMAR VA score − baseline logMAR VA). All VA measurements were evaluated in logMAR.

^**¶**^Spearman correlation coefficient.

*logMAR* logarithm of the minimum angle of resolution, *NFVA* near functional visual acuity, *SVC* Smart Vision Check, *VA* visual acuity, *VMR* visual maintenance ratio.

### Accuracy and reliability of the Smart Vision Check

**Figure 2:**
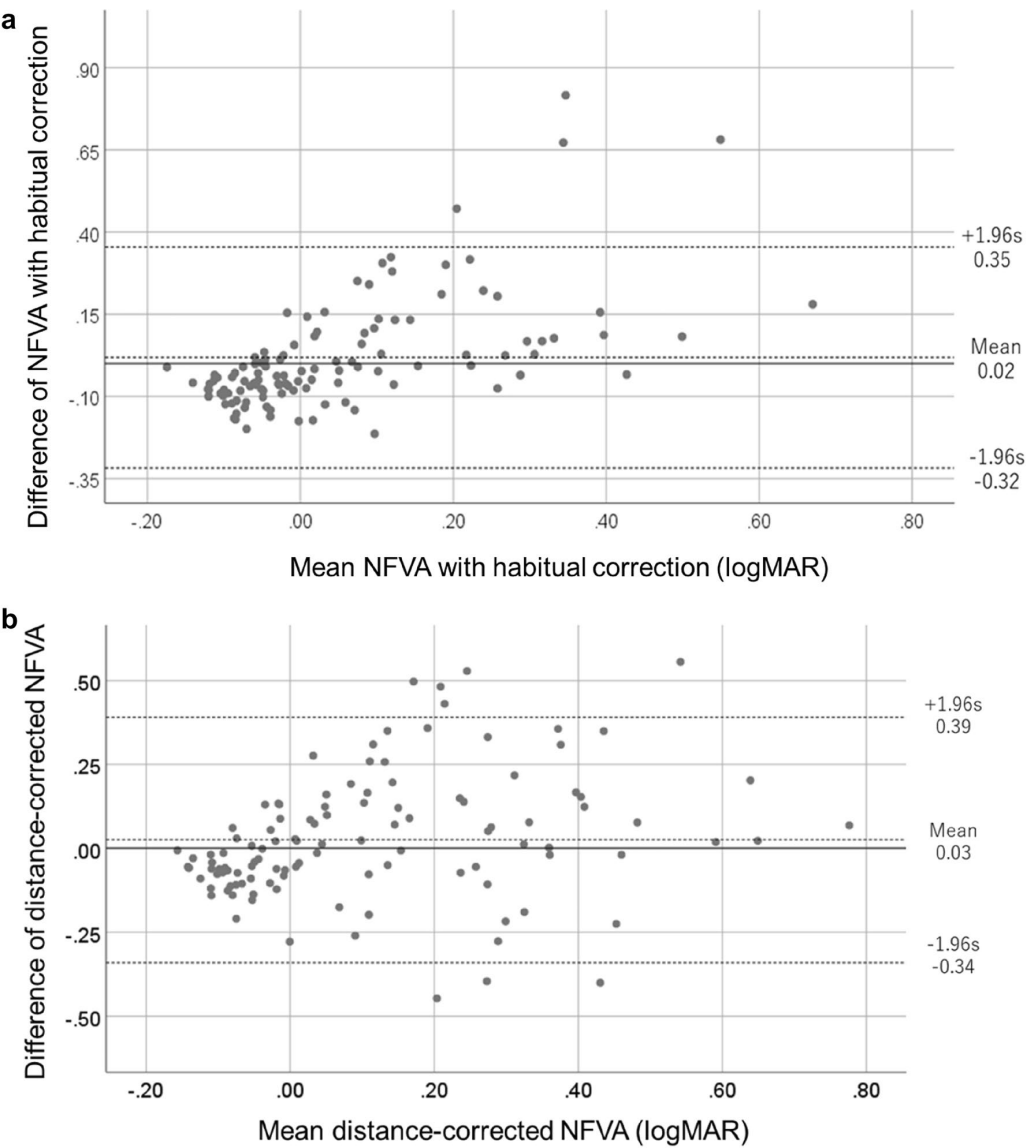
Bland–Altman plots for the averaged (**a**) NFVA with habitual correction and (**b**) distance-corrected NFVA. Bland–Altman plot of the differences in the NFVA with habitual correction (logMAR) between the AS-28 and the SVC. The x-axis represents the mean of the average NFVA between the AS-28 and the SVC, and the y axis represents the mean difference of the two measurements. The mean difference in logMAR acuity (A, 0.02 logMAR; B, 0.03 logMAR) is represented by the solid line and the dashed lines represent the limits of agreement (mean difference ± standard deviation). *logMAR* logarithm of the minimum angle of resolution, *NFVA* near functional visual acuity, *SVC* Smart Vision Check.

When comparing between the AS-28 and the SVC, the Bland–Altman plot indicated that the mean difference (mean bias) for NFVA with habitual correction and distance-corrected NFVA were 0.02 (95% confidence interval [CI] − 0.01 to 0.05) and 0.03 (95% CI − 0.01 to 0.06), respectively, where the mean bias was not significantly different from 0.00 logMAR, as indicated by the inclusion of the x-axis within the CIs (Fig. 2a,b). Limits of agreements (LOAs) for NFVA with habitual correction and distance-corrected NFVA lay on average at ± 0.34 logMAR and ± 0.37 logMAR, respectively, in which the majority of the participants lay within the LOAs. The intraclass correlation coefficients (ICCs) of the repeated SVC measurements was 0.915 (95% CI 0.800–0.969).

## Discussion

In this study, we aimed to evaluate the accuracy and reliability of the SVC for measuring NFVA. We did not observe a significant difference in NFVA measurements between the conventional AS-28 and the SVC techniques in the study population. The measurement of other NFVA-related parameters, such as maximal and minimal FVA, average response time, and visual maintenance ratio (VMR), were generally comparable between the AS-28 and the SVC in the pooled analysis. In addition, we observed a good correlation between the AS-28 and the SVC with excellent reproducibility (> 0.90), indicating the efficacy of the SVC for self-monitoring visual function.

Because of their multifunctionality and flexibility, mobile health technologies are increasingly used in medical practices^11–13^. Due to the proximity and complexity of ophthalmic examinations, patients with non-emergent diseases encounter severely limited access to healthcare services during the COVID-19 pandemic. Therefore, a method for at-home assessment of visual function is urgently required. Nonetheless, detecting presbyopia is generally problematic because (1) measuring VA at a specific moment fails to reflect the daily visual performance^9^ and (2) majority of patients are unaware of their symptoms^5^. Monitoring a dynamic change in vision-related indices in a portable device, the SVC is useful for raising awareness and preventing the exacerbation of presbyopia, regardless of the participants’ recognition.

To the best of our knowledge, we are the first to develop a smartphone-based application to evaluate near visual function. This application is useful because NFVA reflects more sharply reduced visual function than conventional VA testing even in those with early stage presbyopia^10^. In our previous study, decreased accommodative amplitude (proxy for presbyopia) was more strongly related to NFVA than to near VA as measured by conventional VA testing (NFVA: r = − 0.681; conventional near VA: r = − 0.507, P < 0.001) among those with presbyopia^10^. Furthermore, a linear regression analysis indicated that the effect estimate per 1.00 D decrease in accommodative power was greater in NFVA (− 0.075 ± 0.013) than in near VA (− 0.050 ± 0.011)^10^. In this study, we found good correlations evaluating both NFVA with habitual correction and distance-corrected NFVA between the AS-28 and the SVC (NFVA with habitual correction: r = 0.62, P < 0.001; distance-corrected NFVA: r = 0.66, P < 0.001). Additionally, there were no significant differences between the AS-28 and the SVC in the pooled analysis (P > 0.05). Collectively, this application provides similar functionality to that of existing FVA testing, thus showing potential for detecting early presbyopia.

Compared to the AS-28, the SVC assessment had minimal bias (a mean difference of 0), with LOAs of ± 0.34 logMAR for NFVA with habitual correction and ± 0.37 logMAR for NFVA with distance correction. The LOAs in our results had relatively high variation compared to those reported using other smartphone-based VA testing applications. For instance, the Kay iSight Test Professional App (Kay Pictures Ltd., Hertfordshire, United Kingdom), one of the most efficient mobile VA testing apps^17^, reported LOAs between application and clinical measurements of ± 0.414 logMAR^18^. The LOAs between application and clinical measurements from other peer-reviewed research include ± 0.125–0.208 logMAR for Peek Acuity (Peek Vision Ltd, London, United Kingdom)^19^, 0.198 logMAR for GoCheck Kids (Gobiquity, lnc., Arizona, USA)^20^, and ± 0.174 logMAR for DigVis (Cambridge Medical Innovation Ltd., Essex, England)^21^. The SVC is not fully comparable with other VA applications because FVA measures dynamic changes in VA over time, and thus should fluctuate more among different platforms. Nonetheless, the SVC has a potential to evaluate NFVA obtained using the conventional AS-28 with no evidence of bias, suggesting the clinical applicability of this application; however, more extensive validation studies are needed to determine its practical use.

To examine the efficacy of the SVC for evaluating certain populations, we performed several stratified analyses. In general, no substantial difference in NFVA measurements was found between the SVC and the AS-28 when stratified by sex, TBUT measurement, or use of contact lenses. However, a marginally significant difference was found when stratified by age, where NFVA with habitual correction as measured by the SVC was better than that measured by the AS-28 in an elderly population (≥ 43 years old, median value of this population): 0.22 ± 0.20 logMAR with the AS-28, and 0.15 ± 0.14 logMAR with the SVC (P = 0.04). Furthermore, both NFVA with habitual correction and distance-corrected NFVA were significantly better when measured with the AS-28 than when measured with the SVC among those with particularly good NVA (NVA of < − 0.10 logMAR). The mean NFVA with habitual correction was − 0.15 ± 0.02 when measured with the AS-28 and − 0.06 ± 0.07 when measured with the SVC (P < 0.001), whereas distance-corrected NFVA was − 0.15 ± 0.02 when measured with the AS-28 and − 0.06 ± 0.05 when measured with the SVC (P < 0.001). One plausible reason for the discrepancy between the AS-28 and the SVC may be the different resolution of the monitor. While the iPhone SE2 display was limited to 326 ppi (digital image), AS-28 used analogue optical lenses that provided clearer optotype image than the SVC, leading to a significant difference when reading smallest optotypes. In a clinical study of 100 participants aged 18–89 years comparing the NVA measured by smartphone-VA testing applications and conventional near vision card, the application significantly overestimated the NVA relative to that indicated by the near vision card by an average of 0.11 logMAR^22^, suggesting a potential disparity in VA measurement using different techniques with high contrast and brightness levels. This may also explain the marginal difference by age noted in the present study because older participants have poorer contrast sensitivity partly due to cataract, and thus achieve a poorer NFVA on the SVC than on the AS-28, because of the lower contrast on the SVC. Due to the limited number of pixels on the iPhone display, the Landolt ring widths with NFVA smaller than − 0.10 logMAR may not be discernible on the display, and the SVC may underestimate NFVA for those with NFVA < − 0.10 logMAR. Therefore, our data should be interpreted with caution.

The SVC has notable strengths. This application is the first validated smartphone-based NFVA test, which enabled us to detect the dynamic change of VA that is closely linked to the ocular surface condition and quality of life. All tests were fully automated and did not require clinician input unless taking care of the right viewing distance of 40 cm. Furthermore, this application can identify asymptomatic early presbyopia, thereby providing the possibility to reduce health disparities and increase patient outcomes related to difficulty in near vision work. If the SVC is applied in practice, this application may be used by a diverse population in a large sample size at a relatively low-cost.

However, this study has some limitations. First, our study sample was limited, and we cannot eliminate the possibility that a difference between the AS-28 and the SVC was not detected due to the lack of statistical power. Nonetheless, based on similar previous studies, we conducted a power calculation and identified that n = 115 should be adequate to detect a difference (if it exists). Second, the age distribution of the sampled population was weighted toward 30–40-year-olds and only three participants were in their 60 s. Because the recruitment period overlapped with the COVID-19 lockdown, the lower proportion of elderly participants reflects the pattern of attendance at eye clinics. In addition, given that this was a pilot study, we limited the number of participants with binocular CDVA of at least 20/25. Hence, further study in participants with poorer VA or those aged > 60 years is required to verify the potential of the SVC. All examinations were conducted under an ophthalmologists’ supervision. In the application, a detailed explanation of the distance measurement was presented, and users were required to hold the iPhone with both hands and operate it on a desk with a test distance of 40 cm, which was measured using rulers. Nonetheless, the current system is still not applicable in the home setting because it lacks accuracy in measuring the viewing distance. An automatic measurement function in this application is essential for public use in the future.

In summary, we developed a new device, the SVC, and demonstrated its applicability for self-monitoring NFVA in a relatively healthy population. The SVC can evaluate NFVA-related parameters comparable to conventional FVA tests, with high reproducibility. Harnessing the benefits of telemedicine, we believe that the SVC may improve our understanding of the daily changes in NFVA and related at-risk individuals, thereby potentially screening presbyopia at an early stage.

## Methods

### Setting and participants

We conducted a clinic-based, prospective observational study at the Shonan Keiiku Hospital (Kanagawa, Japan) and Keio University School of Medicine (Tokyo, Japan). We posted the study participation invitations and information on our department website and recruited participants between December 17, 2020 and December 9, 2021. In this study, the study participants were solely recruited for research purposes because this study was conducted during the COVID-19 pandemic and we could not recruit enough patients. Therefore, most of the participants were health care providers in these institutes. The study protocol was approved by the respective institutional review boards (approval numbers: 2020-K-8 for Shonan Keiiku Hospital; 20200157 for Keio University School of Medicine), which followed the tenets of the Declaration of Helsinki. All participants provided written informed consent prior to participation. The study protocol was registered with the University Hospital Medical Information Network Clinical Trial Registry (UMIN 000041819).

A total of 115 participants were recruited for this study. We validated the sample size based on the previous literature, which required a sample size of n = 28 to achieve a 90% power with significance level of 0.05 to detect a minimum difference of 0.17 logMAR using a paired-sample t-test^23^.

The inclusion criteria were as follows: age ≥ 20 years, no history of ocular laser treatment or surgery including refractive or cataract surgeries, and a binocular CDVA of at least 20/25. Participants were excluded if they had cognitive disability; severe dry eye disease (defined as presence of dry eye symptoms and an OSS score of ≥ 3 according to the van Bijsterveld scoring system)^24^ because FVA is significantly decreased in these patients^25^; or required interpreting services on their iPhone device.

### Near functional visual acuity measurement using the AS-28 (conventional device)

FVA was measured using the AS-28 visual acuity tester (Kowa, Aichi, Japan), a peeping-type system under full refractive correction and spontaneous blinking without topical anaesthesia. The details of the AS-28 protocols have been described elsewhere^8^. Briefly, this device measures time-wise changes in continuous VA during the test. The Landolt optotypes are presented in the device, and their sizes change depending on the correctness of the responses. In this study, we set the measurement time to 60 s and the display time to 2 s. By handling the joystick, the participants responded to the orientation of the automatically presented Landolt ring. FVA was evaluated using five parameters: average, maximal, and minimal VA; average response time; and VMR. FVA (known as ‘average VA’) is defined as the mean value of the time-wise change in the continuous VA during the test (60 s). The VMR is the ratio of FVA divided by the best-corrected Landolt VA (baseline VA). The maximal and minimal VAs are defined as the highest and lowest VAs recorded during the examination. To measure NFVA, all the participants underwent FVA testing with -2.50 D added to the best distance correction at baseline.

### Near functional visual acuity measurement using the Smart Vision Check

**Figure 3:**
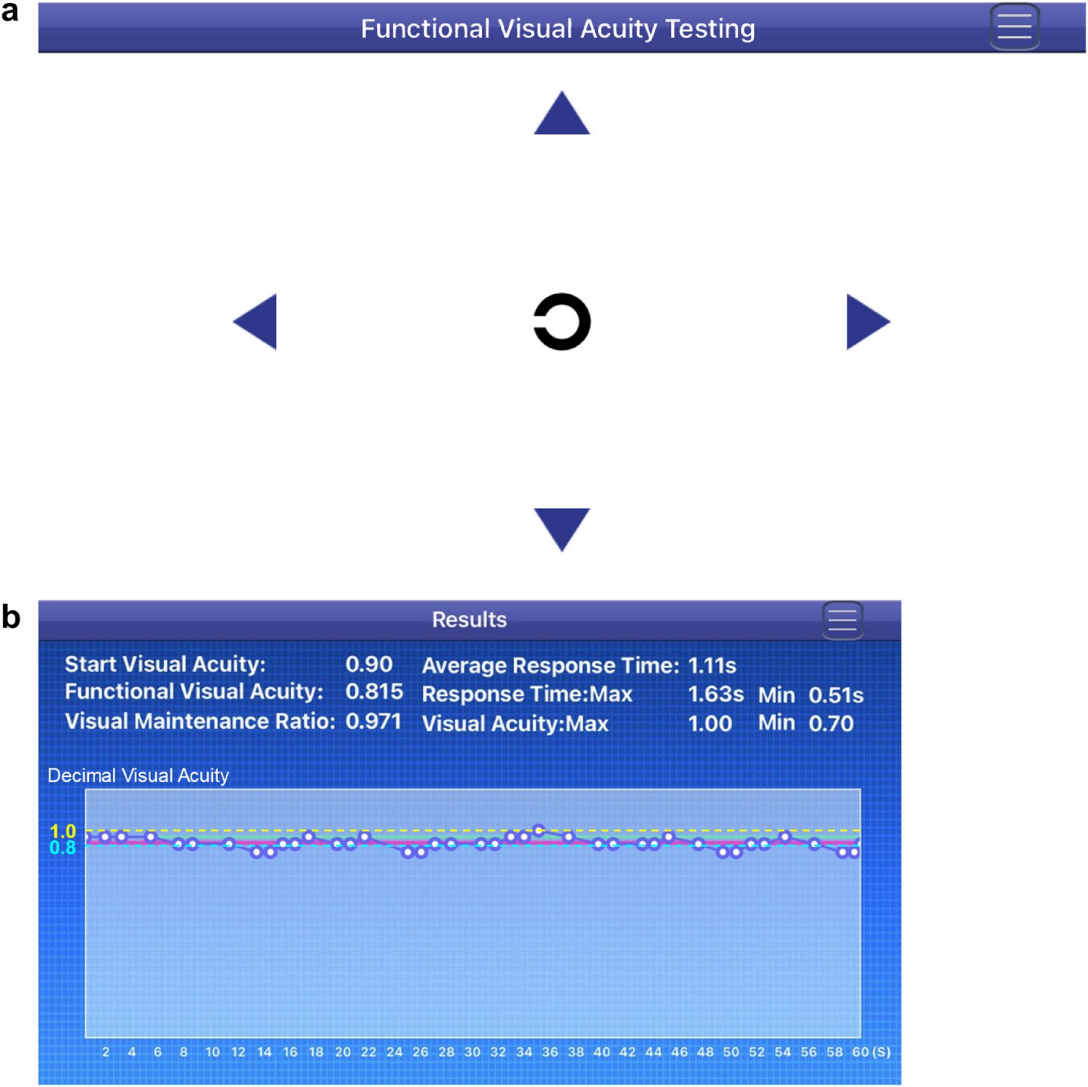
Display of the Smart Vision Check (SVC). The x-axis represents the screen time and the y-axis represents decimal VA. The yellow and blue dotted lines indicate VA of 1.0 and 0.8, respectively. The green and pink solid lines indicate the start VA and NFVA, respectively. (**a**) The SVC displays the optotype and the participants were required to delineate the orientation of the automatically presented Landolt ring on the screen by tapping on the orientation icon within 2 s. (**b**) The SVC test takes 60 s in total and automatically evaluates the five parameters related to NFVA including maximal and minimal VA, average response time, and VMR. *NFVA* near functional visual acuity, *SVC* Smart Vision Check, *VA* visual acuity, *VMR* visual maintenance ratio.

The SVC application is designed to be uploaded and used on an Apple iPhone SE2 (Apple Inc., 2020) to flexibly measure NFVA. The iPhone SE2 has screen dimensions of 138.4 × 67.3 mm and a pixel resolution of 750 × 1,334 pixels resulting in a resolution of 326 ppi. The SVC consists of a computing device and a display. Before use, the participants were required to hold the smartphone in front at a distance of 40 cm from the eyes at full brightness while wearing previously obtained corrective lenses for near vision (if any). The trained ophthalmologic technician measured the distance between the phone and the participants’ eye using a tape metre at the beginning of the test and supervised it throughout the test to avoid any changes. When the participants tapped the start button, the SVC displayed the optotype (Fig. 3a). The participants were required to delineate the orientation of the automatically presented Landolt ring on the iPhone screen by tapping on the orientation icon within 2 s. No response within 2 s was regarded as incorrect. The optotype automatically increases by one size in decimal VA when the response was incorrect or when there was no response. When the participants responded correctly, an optotype of the same size was displayed at random again. The optotype decreases by one size in decimal VA when the next answer was correct. The SVC test takes 60 s in total and automatically evaluates the five NFVA parameters (Fig. 3b). In the statistical analysis, the decimal values were converted to the logMAR units.

### Other ocular examinations

All ophthalmic examinations were performed by trained ophthalmic technicians, except for the TBUT and OSS scores, which were measured by board-certified ophthalmologists. Monocular CDVA, monocular DCNVA, binocular CDVA, binocular DCNVA, binocular DVAHC, and binocular NVAHC were measured according to the Japanese Industrial Standards^26^. We used a decimal VA chart and then converted the decimal values to the logMAR units for statistical analysis. The amplitude of accommodation and pupillary diameters were measured using an auto kerato-refractometer (ARK-1s; NIDEK Co., Ltd., Aichi, Japan). According to the Japanese dry eye diagnostic criteria^27^, subjective dry eye symptoms (dry sensation, foreign-body sensation, ocular pain, ocular fatigue, sensitivity to bright light, and blurred vision) were obtained from self-reported questionnaires. After instillation of fluorescein sodium in the conjunctival sac using a test paper (Ayumi Pharmaceutical Corp., Tokyo, Japan), the interval between the last complete blink and the appearance of the first black corneal spot on the stained tear film was measured as the TBUT three times, and the mean value was calculated. A cobalt blue filter was used to measure the TBUT. A TBUT of ≤ 5 s was considered abnormal. The van Bijsterveld OSS scoring system was used, wherein the ocular surface was divided into three zones, namely, nasal conjunctival, corneal, and temporal conjunctival, and scored on a 0–3-point scale in each zone (0: no damage to 3: damaged entirely)^23^. Each score was then summed for a maximum total score of 9 points.

### Statistical analysis

Analysis and data visualisation were performed using SAS (Version 9.4, SAS Institute, Cary, NC, USA) and SPSS (IBM SPSS Statistics, version 27, Armonk, NY, USA). All significance tests were two-sided, and the significance level was set at α = 0.05. Baseline characteristics are presented as counts and proportions for categorical variables and means ± standard deviation for continuous values.

Measurements of the five NFVA parameters using the conventional AS-28 and the SVC were compared using a paired t-test and Spearman’s rank correlation coefficients. To evaluate the level of agreement between the SVC and AS-28 NFVA measurements, we used the Bland–Altman plots to visualise the 95% LOAs and mean bias. The test–retest agreement was evaluated using mixed-effect model two-way ICCs.

In the secondary analyses, we performed a paired t-test to examine whether the NFVA measurements differed between the AS-28 and the SVC in certain populations (those with previously known risk factors for decreased FVA). As FVA may have high intra-variability among those with dry eye disease, it is necessary to assess whether the FVA is comparable among these two methods. Therefore, stratification analyses were performed by age (< 43 vs. ≥ 43 years old; cut-off point for the median value of age in the study participants), sex (men vs. women), the presence of clinical dry eye signs (TBUT of < 5 vs. ≥ 5 s), use of contact lenses (yes vs. no), and NVA (< − 0.10 vs. ≥ − 0.10 logMAR).

## Supplementary Information

Supplementary Information.

## Data Availability

The datasets generated during and/or analysed during the current study are available from the corresponding author on reasonable request.
