# Supplementary material for: Validation of a novel iPhone application for evaluating near functional visual acuity
Source: Sci Rep. 2022 Dec 26;12:22342. doi: 10.1038/s41598-022-27011-2 (PMC9791149; doi:10.1038/s41598-022-27011-2)
Supplement: Supplementary file 1 — Supplementary Information. [file 41598_2022_27011_MOESM1_ESM.docx]

**Supplementary materials**

**Validation of a novel iPhone application for evaluating near functional visual acuity**

**Co-corresponding Authors:**

Akiko Hanyuda, MD, PhD, MPH

Department of Ophthalmology, Keio University School of Medicine,

35 Shinanomachi, Shinjuku-ku, Tokyo 160-8582, Japan

Telephone: +81-3-3353-1211

E-mail: [akihanyu@keio.jp](about:blank)

Kazuno Negishi, MD, PhD

Department of Ophthalmology, Keio University School of Medicine,

35 Shinanomachi, Shinjuku-ku, Tokyo 160-8582, Japan

Telephone: +81-3-3353-1211

E-mail: [kazunonegishi@keio.jp](about:blank)

**Table S1.** Measurement of near functional visual acuity (NFVA)^a^ by the AS-28 and the Smart Vision Check (SVC)^a^ in the strata of age, sex, the presence of dry eye, measurement of NFVA, and use of contact lenses

| **Parameters** | **AS-28** | **SVC** | ***P* Value^b^** |
| --- | --- | --- | --- |
| ***Age < 43 (n=60)*** |  |  |  |
| **NFVA with habitual correction** |  |  |  |
| Average VA | -0.05 (0.12) | -0.03 (0.05) | 0.21 |
| Maximal VA | -0.14 (0.11) | -0.13 (0.11) | 0.45 |
| Minimal VA | 0.07 (0.16) | 0.07 (0.06) | 0.95 |
| Average response time | 1.28 (0.16) | 1.26 (0.12) | 0.30 |
| VMR | 0.96 (0.06) | 0.94 (0.04) | 0.02 |
| **Distance-corrected NFVA** |  |  |  |
| Average VA | -0.01 (0.17) | -0.03 (0.06) | 0.36 |
| Maximal VA | -0.11 (0.13) | -0.13 (0.06) | 0.29 |
| Minimal VA | 0.12 (0.25) | 0.07 (0.10) | 0.13 |
| Average response time | 1.28 (0.17) | 1.26 (0.13) | 0.31 |
| VMR | 0.95 (0.08) | 0.94 (0.03) | 0.62 |
| ***Age ≥ 43 (n=55)*** |  |  |  |
| **NFVA with habitual correction** |  |  |  |
| Average VA | 0.22 (0.20) | 0.15 (0.14) | 0.04 |
| Maximal VA | 0.06 (0.20) | 0.02 (0.20) | 0.17 |
| Minimal VA | 0.42 (0.30) | 0.33 (0.20) | 0.17 |
| Average response time | 1.31 (0.14) | 1.30 (0.11) | 0.33 |
| VMR | 0.90 (0.09) | 0.90 (0.06) | 0.58 |
| **Distance-corrected NFVA** |  |  |  |
| Average VA | 0.27 (0.23) | 0.24 (0.19) | 0.27 |
| Maximal VA | 0.13 (0.20) | 0.08 (0.15) | 0.03 |
| Minimal VA | 0.50 (0.31) | 0.47 (0.27) | 0.48 |
| Average response time | 1.29 (0.14) | 1.31 (0.09) | 0.20 |
| VMR | 0.90 (0.11) | 0.90 (0.09) | 0.65 |
| ***Men (n=73)*** |  |  |  |
| **NFVA with habitual correction** |  |  |  |
| Average VA | 0.07 (0.21) | 0.05 (0.13) | 0.28 |
| Maximal VA | -0.06 (0.18) | -0.07 (0.12) | 0.53 |
| Minimal VA | 0.21 (0.27) | 0.18 (0.19) | 0.14 |
| Average response time | 1.30 (0.15) | 1.29 (0.13) | 0.71 |
| VMR | 0.93 (0.09) | 0.93 (0.05) | 0.67 |
| **Distance-corrected NFVA** |  |  |  |
| Average VA | 0.12 (0.24) | 0.11 (0.21) | 0.52 |
| Maximal VA | 0.01 (0.21) | -0.02 (0.15) | 0.08 |
| Minimal VA | 0.31 (0.32) | 0.27 (0.30) | 0.34 |
| Average response time | 1.30 (0.16) | 1.28 (0.12) | 0.35 |
| VMR | 0.92 (0.11) | 0.92 (0.07) | 0.80 |
| ***Women (n=42)*** |  |  |  |
| **NFVA with habitual correction** |  |  |  |
| Average VA | 0.05 (0.24) | 0.03 (0.13) | 0.62 |
| Maximal VA | -0.07 (0.18) | -0.08 (0.11) | 0.76 |
| Minimal VA | 0.19 (0.29) | 0.19 (0.20) | 0.94 |
| Average response time | 1.29 (0.14) | 1.29 (0.12) | 0.83 |
| VMR | 0.96 (0.06) | 0.92 (0.07) | 0.01 |
| **Distance-corrected NFVA** |  |  |  |
| Average VA | 0.12 (0.27) | 0.08 (0.19) | 0.12 |
| Maximal VA | 0.00 (0.23) | -0.04 (0.16) | 0.11 |
| Minimal VA | 0.29 (0.36) | 0.24 (0.24) | 0.19 |
| Average response time | 1.25 (0.15) | 1.29 (0.11) | 0.22 |
| VMR | 0.93 (0.09) | 0.92 (0.07) | 0.33 |
|  |  |  |  |
|  |  |  |  |
| ***TBUT*** ***≤ 5 s (n=44)*** |  |  |  |
| **NFVA with habitual correction** |  |  |  |
| Average VA | 0.07 (0.24) | 0.05 (0.12) | 0.37 |
| Maximal VA | -0.04 (0.21) | -0.07 (0.10) | 0.33 |
| Minimal VA | 0.22 (0.29) | 0.18 (0.16) | 0.24 |
| Average response time | 1.34 (0.14) | 1.32 (0.14) | 0.55 |
| VMR | 0.92 (0.10) | 0.92 (0.06) | 0.92 |
| **Distance-corrected NFVA** |  |  |  |
| Average VA | 0.13 (0.25) | 0.11 (0.19) | 0.49 |
| Maximal VA | 0.00 (0.21) | -0.03 (0.13) | 0.30 |
| Minimal VA | 0.30 (0.34) | 0.28 (0.27) | 0.56 |
| Average response time | 1.33 (0.25) | 1.31 (0.13) | 0.57 |
| VMR | 0.91 (0.12) | 0.91 (0.06) | 0.94 |
| ***TBUT > 5 s (n=71)*** |  |  |  |
| **NFVA with habitual correction** |  |  |  |
| Average VA | 0.05 (0.22) | 0.04 (0.13) | 0.48 |
| Maximal VA | -0.07 (0.15) | -0.07 (0.12) | 0.94 |
| Minimal VA | 0.20 (0.27) | 0.19 (0.21) | 0.69 |
| Average response time | 1.27 (0.14) | 1.28 (0.11) | 0.71 |
| VMR | 0.95 (0.06) | 0.93 (0.05) | 0.005 |
| **Distance-corrected NFVA** |  |  |  |
| Average VA | 0.12 (0.25) | 0.09 (0.20) | 0.17 |
| Maximal VA | 0.01 (0.21) | -0.03 (0.16) | 0.02 |
| Minimal VA | 0.30 (0.34) | 0.25 (0.29) | 0.12 |
| Average response time | 1.26 (0.15) | 1.27 (0.11) | 0.40 |
| VMR | 0.93 (0.09) | 0.92 (0.08) | 0.44 |
| ***NVA < -0.10 logMAR (n=18)*** |  |  |  |
| **NFVA with habitual correction** |  |  |  |
| Average VA | -0.15 (0.02) | -0.06 (0.07) | <.001 |
| Maximal VA | -0.18 (0.00) | -0.15 (0.04) | <.001 |
| Minimal VA | -0.06 (0.7) | 0.06 (0.07) | <.001 |
| Average response time | 1.26 (0.18) | 1.22 (0.12) | 0.39 |
| VMR | 1.00 (0.02) | 0.94 (0.03) | <.001 |
| **Distance-corrected NFVA** |  |  |  |
| Average VA | -0.15 (0.02) | -0.06 (0.05) | <.001 |
| Maximal VA | -0.18 (0.00) | -0.14 (0.05) | <.001 |
| Minimal VA | -0.05 (0.06) | 0.04 (0.07) | <.001 |
| Average response time | 1.23 (0.15) | 1.23 (0.12) | 0.90 |
| VMR | 1.00 (0.01) | 0.95 (0.03) | <.001 |
| ***NVA ≥ -0.10 logMAR (n=97)*** |  |  |  |
| **NFVA with habitual correction** |  |  |  |
| Average VA | 0.10 (0.22) | 0.06 (0.13) | 0.47 |
| Maximal VA | -0.04 (0.19) | -0.06 (0.12) | 0.28 |
| Minimal VA | 0.25 (0.28) | 0.20 (0.20) | 0.19 |
| Average response time | 1.30 (0.14) | 1.31 (0.12) | 0.82 |
| VMR | 0.93 (0.08) | 0.92 (0.06) | 0.47 |
| **Distance-corrected NFVA** |  |  |  |
| Average VA | 0.17 (0.23) | 0.13 (0.20) | 0.04 |
| Maximal VA | 0.04 (0.21) | -0.01 (0.16) | 0.01 |
| Minimal VA | 0.36 (0.33) | 0.30 (0.29) | 0.04 |
| Average response time | 1.30 (0.16) | 1.30 (0.12) | 0.89 |
| VMR | 0.91 (0.11) | 0.91 (0.08) | 0.95 |
| ***Contact lens users (n=34)*** |  |  |  |
| **NFVA with habitual correction** |  |  |  |
| Average VA | 0.02 (0.16) | 0.02 (0.11) | 0.98 |
| Maximal VA | -0.08 (0.14) | -0.09 (0.14) | 0.78 |
| Minimal VA | 0.16 (0.22) | 0.14 (0.16) | 0.45 |
| Average response time | 1.29 (0.14) | 1.27 (0.13) | 0.28 |
| VMR | 0.95 (0.06) | 0.93 (0.05) | 0.23 |
|  |  |  |  |
|  |  |  |  |
| ***Contact lens users (n=34)*** |  |  |  |
| **Distance-corrected NFVA** |  |  |  |
| Average VA | 0.06 (0.21) | 0.08 (0.20) | 0.71 |
| Maximal VA | -0.04 (0.19) | -0.05 (0.14) | 0.77 |
| Minimal VA | 0.21 (0.30) | 0.23 (0.26) | 0.77 |
| Average response time | 1.26 (0.14) | 1.27 (0.12) | 0.15 |
| VMR | -0.04 (0.19) | -0.05 (0.13) | 0.71 |
| ***Non-users of contact lenses (n=81)*** |  |  |  |
| **NFVA with habitual correction** |  |  |  |
| Average VA | 0.08 (0.25) | 0.05 (0.14) | 0.22 |
| Maximal VA | -0.05 (0.19) | -0.06 (0.12) | 0.53 |
| Minimal VA | 0.22 (0.30) | 0.20 (0.21) | 0.40 |
| Average response time | 1.30 (0.15) | 1.30 (0.12) | 0.58 |
| VMR | 0.94 (0.08) | 0.92 (0.06) | 0.18 |
| **Distance-corrected NFVA** |  |  |  |
| Average VA | 0.15 (0.26) | 0.11 (0.20) | 0.10 |
| Maximal VA | 0.02 (0.22) | -0.02 (0.16) | 0.01 |
| Minimal VA | 0.34 (0.35) | 0.27 (0.29) | 0.05 |
| Average response time | 1.29 (0.17) | 1.29 (0.12) | 0.92 |
| VMR | 0.92 (0.11) | 0.92 (0.08) | 0.80 |

^a^ The NFVA was evaluated by five parameters, including average, maximal, and minimal Vas; average response time; and VMR. The VMR is the ratio of FVA divided by the value of baseline VA: VMR= (lowest logMAR VA score − logMAR FVA) / (lowest logMAR VA score − baseline logMAR VA). All VA measurements were evaluated in logMAR.
^b^Paired t-test.

Abbreviations: logMAR, logarithm of the minimum angle of resolution; NFVA, near functional visual acuity; SVC, Smart Vision Check; VA, visual acuity; VMR, visual maintenance ratio.
